# Association between cardiovascular health measured by Life’s Essential 8 and depressive symptoms

**DOI:** 10.4178/epih.e2026013

**Published:** 2026-02-27

**Authors:** Jeong Hyun Ahn, Hyejin Kim, Hyeon Chang Kim, Hokyou Lee, Younga Heather Lee, Donald M. Lloyd-Jones, Sun Jae Jung

**Affiliations:** 1 Department of Public Health, Graduate School, Yonsei University, Seoul, Korea; 2 Department of Preventive Medicine, Yonsei University College of Medicine, Seoul, Korea; 3 Institute for Innovation in Digital Healthcare, Yonsei University, Seoul, Korea; 4 Psychiatric & Neurodevelopmental Genetics Unit, Center for Genomic Medicine, Massachusetts General Hospital, Boston, MA, USA; 5 Department of Psychiatry, Harvard Medical School, Boston, MA, USA; 6 Broad Trauma Initiative, Broad Institute of MIT and Harvard, Cambridge, MA, USA; 7 Section of Preventive Medicine and Epidemiology, Department of Medicine, Boston University Chobanian & Avedisian School of Medicine, Boston, MA, USA

**Keywords:** Life’s Essential 8, Cardiovascular health, Health behaviors, Depressive symptoms

## Abstract

**OBJECTIVES:**

Poor cardiovascular health (CVH) and the high prevalence of depressive symptoms represent significant public health concerns, underscoring the importance of examining their association. This study aimed to investigate the association between CVH, as defined by the American Heart Association’s 2022 Life’s Essential 8 (LE8) framework, and depressive symptoms.

**METHODS:**

This study used data from the 2014, 2016, 2018, and 2020 Korea National Health and Nutrition Examination Survey. Overall CVH, measured using LE8, was categorized into 3 groups: low score (0–<50), moderate score (50–<80), and high score (80–100). LE8 comprises 2 subdomains: health behaviors and health factors. Depressive symptoms were defined as a total score ≥10 on the Patient Health Questionnaire-9. Multiple logistic regression analyses were performed, adjusting for sex, age, socioeconomic status, and current drinking status.

**RESULTS:**

Among 17,294 adults, 257 male and 681 female reported significant depressive symptoms. Compared with individuals in the low LE8 category (reference), the odds ratio (OR) for depressive symptoms was 0.29 (95% confidence interval [CI], 0.21 to 0.40) for those in the high LE8 category. The OR for depressive symptoms was 0.39 (95% CI, 0.29 to 0.53) for individuals with a high health behavior score compared with those with a low health behavior score. In contrast, the health factor score was not significantly associated with depressive symptoms.

**CONCLUSIONS:**

These findings suggest that overall CVH, particularly the health behavior subdomain, was associated with lower odds of depressive symptoms. Prospective longitudinal studies are warranted to validate these findings and clarify the directionality of the observed associations.

## GRAPHICAL ABSTRACT

[Fig f5-epih-48-e2026013]

## Key Message

This study used nationally representative data to examine the association between cardiovascular health (CVH), measured by Life’s Essential 8, and depressive symptoms among Korean adults. Individuals with higher CVH scores indicated more ideal cardiovascular health. Participants with moderate or high CVH were associated with lower odds of depressive symptoms compared with low CVH. Moreover, similar associations were observed for individual CVH components, including ideal diet, non-smoking status, ideal sleep health, and ideal blood glucose. These findings warrant further investigation using longitudinal study designs.

## INTRODUCTION

Depression is a common mental health disorder worldwide and a leading contributor to mortality [1,2]. According to the Global Burden of Disease Study, the number of individuals with depression worldwide increased by 0.59% in 2019 [3]. In Korea, the prevalence of depression or depressive symptoms reached 37% during March 2020–April 2021, markedly higher than in many other countries, highlighting the substantial burden of depression in the Korean population [4]. According to the World Health Organization, cardiovascular diseases cause millions of deaths worldwide each year [5].

In 2010, the American Heart Association (AHA) developed the “Life’s Simple 7” (LS7) metrics to assess cardiovascular health (CVH) and promote population health [6]. A systematic review reported that 5 studies examined the association between LS7-measured CVH and depressive symptoms [7]. A prospective study of 5,110 United States adults and another study involving 9,214 participants in Brazil both found that higher LS7 scores were associated with lower odds of depressive symptoms [8,9]. However, limitations have been noted in LS7 quantification for population-level CVH, particularly regarding diet metrics and the omission of sleep as a component [10,11].

For these reasons, in 2022, the AHA revised the CVH metrics and introduced “Life’s Essential 8” (LE8), incorporating sleep health and providing more detailed definitions for each metric [10]. Sleep health, a newly added component of CVH, has been widely reported to be associated with depressive symptoms through multiple mechanisms, including the neuroendocrine stress system and serotonin regulation [12]. A systematic review and meta-analysis demonstrated that higher LE8 scores were associated with reduced risks of cardiovascular disease, all-cause mortality, cardiovascular disease-specific mortality, coronary heart disease, and stroke [13].

However, few studies have examined the association between LE8-defined CVH and depressive symptoms. Two prospective cohort studies using UK Biobank data reported that CVH measured by LE8 was related to depression risk [14,15]. However, individual LE8 components were not examined in these studies. Evaluating each component separately is important because the strength of its association with depressive symptoms may differ across metrics. Furthermore, limited evidence is available on associations between CVH measured by LS7 or LE8 and depressive symptoms in Asian populations.

To address this gap, this study aimed to assess the association between CVH measured using LE8 and depressive symptoms. In addition, we examined the association between each individual CVH metric and depressive symptoms.

## MATERIALS AND METHODS

### Data source and study population

This study used data from the Korea National Health and Nutrition Examination Survey (KNHANES) conducted in 2014, 2016, 2018, and 2020. KNHANES is a surveillance system conducted by the Korea Centers for Disease Control and Prevention (KCDC). It employs multistage, stratified cluster sampling and a cross-sectional design to generate nationally representative estimates [16]. Conducted annually, the survey assesses the health and nutritional status of Koreans aged ≥1 year through health interviews, examinations, and nutrition assessments performed by trained professionals [16].

A total of 31,051 adult participants were included in the 2014, 2016, 2018, and 2020 KNHANES. We excluded participants aged <19 years (n=6,071), pregnant females (n=105), and those with fasting duration <8 hours (n=2,195). Among the remaining 22,680 participants (aged ≥19 years), we excluded 5,386 participants with missing LE8 information (n=3,698) or missing information on depressive symptoms (n=1,688). Finally, 17,294 participants were included in the analysis (Supplementary Material 1). To assess reporting quality for this observational study, we followed the Strengthening the Reporting of Observational Studies in Epidemiology (STROBE) guidelines [17].

### Measurement

#### Assessment of cardiovascular health by LE8 [10]

We used a modified version of LE8 as defined by the AHA. LE8 is divided into 2 domains, consisting of health behavior and health factor metrics. The health behavior metrics include diet, physical activity, nicotine exposure, and sleep health. The health factor metrics comprise body mass index (BMI), blood lipids, blood glucose, and blood pressure (BP). Details regarding the definition and scoring of LE8 are provided in Supplementary Material 2.

#### Health behavior

Because this study targeted a Korean population, we assessed the diet metric using the Korean Healthy Eating Index (KHEI) for adults, developed by the KCDC, which evaluates overall dietary quality among Korean adults [18] (details in Supplementary Materials 2 and 3). This approach is consistent with previous studies that assessed overall diet quality using established indices such as the Healthy Eating Index and the KHEI [19,20]. Breakfast intake was assessed based on the weekly frequency reported over the past year, whereas food and nutrient intake were evaluated using a 24-hour dietary recall for the previous day [18]. We assigned scores according to KHEI quintiles based on AHA guidelines [10]. Physical activity was assessed using the Global Physical Activity Questionnaire, which measures physical activity during a typical week across work, transport, and leisure domains [21]. Total weekly moderate-to-vigorous physical activity was calculated in minutes. The nicotine exposure metric was assessed based on current smoking status, with different scores assigned for never-smokers, former smokers, and current smokers (Supplementary Material 2). In addition, scoring accounted for nicotine delivery system use and exposure to secondhand smoke at home. Sleep health was defined as average daily sleep duration, as reported in a self-report questionnaire. In the 2014 and 2020 KNHANES, participants reported daily sleep duration. In the 2016 and 2018 surveys, sleep duration was calculated using reported bedtimes and wake-up times on weekdays and weekends. According to AHA guidelines and other widely accepted recommendations, the optimal sleep duration for adults is 7–<9 hours [10,22,23] (Supplementary Materials 2 and 4).

#### Health factors

BMI was calculated as weight (kg) divided by height squared (m²) among non-pregnant individuals and scored according to World Health Organization guidelines for Asian populations [24,25]. Blood samples were collected from individuals who fasted for 8 hours. The blood lipid metric was non–high-density lipoprotein cholesterol (non–HDL-C). The optimal level of non–HDL-C is <130 mg/dL [26,27]. Blood glucose was assessed using fasting blood glucose or hemoglobin A1c levels, considering diabetes history, medication use, and insulin injection [28]. BP was measured 3 times on the right arm after participants remained seated for 5 minutes during a rest period. BP was calculated as the average of the second and third measurements. Health factor measurements were obtained using standardized equipment; additional details are provided in Supplementary Material 5.

#### Calculation of the overall CVH score

Each CVH metric was scored from 0 points to 100 points. The overall CVH score was calculated as the unweighted average of the eight metric scores. Health behavior and health factor subdomain scores were calculated using the same method. Following AHA guidelines, overall CVH measured using LE8—as well as the health behavior and health factor scores—was categorized as low (0–<50), moderate (50–<80), and high (80–100) [10]. For each CVH metric, participants scoring 100 were classified as ideal (score=100), whereas those scoring <100 were classified as non-ideal (score <100).

### Assessment of depressive symptoms

Depressive symptoms were assessed using the Korean version of the Patient Health Questionnaire-9 (PHQ-9), which is based on diagnostic criteria from the Diagnostic and Statistical Manual of Mental Disorders, 4th edition [29,30]. The PHQ-9 includes 9 items and asks how often participants experienced depressive symptoms during the past 2 weeks [29,30]. Items are scored on a 4-point scale: 0=not at all, 1=several days, 2=more than half the days, and 3=nearly every day [30]. The total PHQ-9 score ranges from 0 to 27 and has been validated with a sensitivity of 88% and specificity of 88% among patients visiting general medical clinics or obstetrics-gynecology clinics [30]. The PHQ-9 has also been validated in the Korean general population (Cronbach’s α=0.79) [31]. Participants with PHQ-9 scores ≥10 were classified as having depressive symptoms [30].

### Covariates

Covariates included sex (male and female), age groups (19–39, 40–59, and ≥60 years), socioeconomic status (income, educational attainment, and marital status) [9], and current drinking status [7,9]. Income was divided into quartiles (<18.4, 18.4 to <35.5, 35.5 to <58.3, and ≥58.3 million Korean won/yr). Educational attainment was categorized as ≤6 years (elementary school or less), 7–9 years (middle school), 10–12 years (high school), and >12 years (college or above). Marital status was grouped as never married, married and living together, married-separate, and divorced/widowed. Current drinking status was classified as “yes” for those who drank once or more per month in the past year and “no” for those who did not drink or drank less than once per month.

### Statistical analysis

We derived integrated survey weights by multiplying one-fourth of each year’s weight. All analyses used survey weights to account for the complex sampling design. General characteristics of the study population were analyzed using chi-square tests and t-tests. Multiple logistic regression analyses were performed to investigate the association between CVH measured by LE8 and depressive symptoms. Restricted cubic splines were used to examine nonlinear associations between overall CVH and depressive symptoms. Odds ratios (ORs) and 95% confidence intervals (CIs) were calculated. All models were adjusted for sex, age, income, educational attainment, marital status, and current drinking status. We divided LE8 into health factor and health behavior subdomains and conducted the same analyses for each subdomain. In addition, we analyzed associations between each LE8 metric and depressive symptoms. Finally, we conducted stratified analyses by sex and age group.

Sensitivity analyses were conducted to assess the robustness of the findings. First, we compared 2 indices (LS7 and LE8) in relation to depressive symptoms. The area under the curve (AUC) was used to compare model performance for LS7 and LE8. Definitions and scoring for LS7 and LE8 are provided in Supplementary Material 6 and Supplementary Material 2. Second, to reduce potential reverse causality inherent in a cross-sectional design, we excluded individuals previously diagnosed with depression. Third, we conducted sensitivity analyses using an alternative PHQ-9 cutoff (≥5) to capture mild depressive symptoms and recalculated ORs and corresponding 95% CIs for associations between LE8- measured CVH and depressive symptoms. Finally, we performed multiple imputation for missing covariates to assess the robustness of the main findings.

Statistical significance was set at p-value <0.05. Statistical analyses were performed using SAS version 9.4 (SAS Institute Inc., Cary, NC, USA).

### Ethics statement

KNHANES VI (2014), VII (2016, 2018), and VIII (2020) were conducted with approval from the Institutional Review Board of the KCDC (IRB No. 2013–12EXP–03–5C, 2018–01–03–P–A, and 2018–01–03–2C–A), except for 2016, which was exempt under the Bioethics Act. Written informed consent was obtained from all participants.

## RESULTS

Table 1 presents general characteristics of the study population by depressive symptom status. Among 17,294 participants, 938 (5.0%) had depressive symptoms. The mean CVH score was 59.8 (95% CI, 58.7 to 61.0) among participants with depressive symptoms and 64.8 (95% CI, 64.4 to 65.1) among those without depressive symptoms.

**Figure 1.**
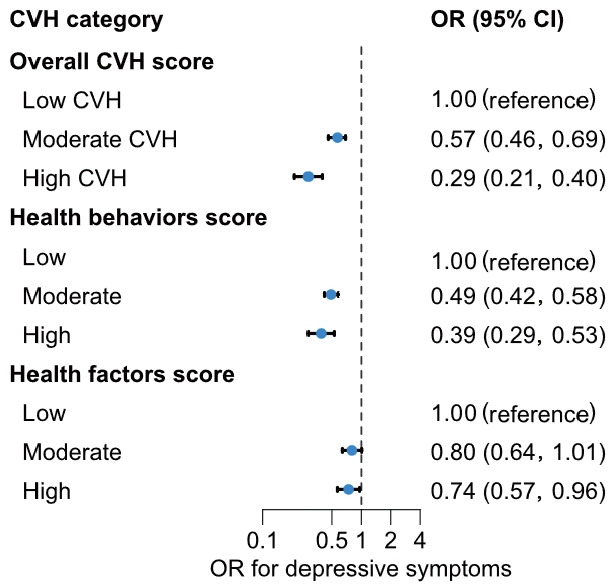
ORs and 95% CIs for the association between CVH scores and depressive symptoms. Overall CVH (by LE8) is divided into two domains: health behaviors (diet, physical activity, nicotine exposure, and sleep health), and health factors (body mass index, blood lipids, blood glucose, and blood pressure). Adjusted for sex, age, income, educational attainment, marital status, and current drinking status. CVH, cardiovascular health; OR, odds ratios; CI, confidence intervals; LE8, Life’s Essential 8.

**Figure 2.**
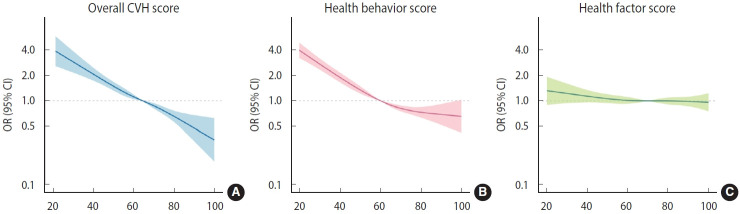
Restricted cubic splines with 95% CIs for the association between CVH using LE8 and depressive symptoms. (A) Overall CVH (by LE8) is divided into two domains: (B) health behaviors (diet, physical activity, nicotine exposure, and sleep health), and (C) health factors (body mass index, blood lipids, blood glucose, and blood pressure). Adjusted for sex, age, income, educational attainment, marital status, and current drinking status. CVH, cardiovascular health; OR, odds ratios; CI, confidence intervals; LE8, Life’s Essential 8.

The association between overall CVH and depressive symptoms is shown in Figure 1. Compared with participants with a low LE8 CVH score, those with moderate CVH (OR, 0.57; 95% CI, 0.46 to 0.69) or high CVH (OR, 0.29; 95% CI, 0.21 to 0.40) had significantly lower odds of depressive symptoms. Compared with participants with low LE8 scores, the OR for depressive symptoms was 0.49 (95% CI, 0.42 to 0.58) among those with a moderate health behavior score and 0.39 (95% CI, 0.29 to 0.53) among those with a high health behavior score. Results from the multiple imputation analyses were consistent with the main findings (Supplementary Material 7). Non-linear associations between health behavior scores and depressive symptoms were observed in spline regression analyses (Figure 2).

**Figure 3.**
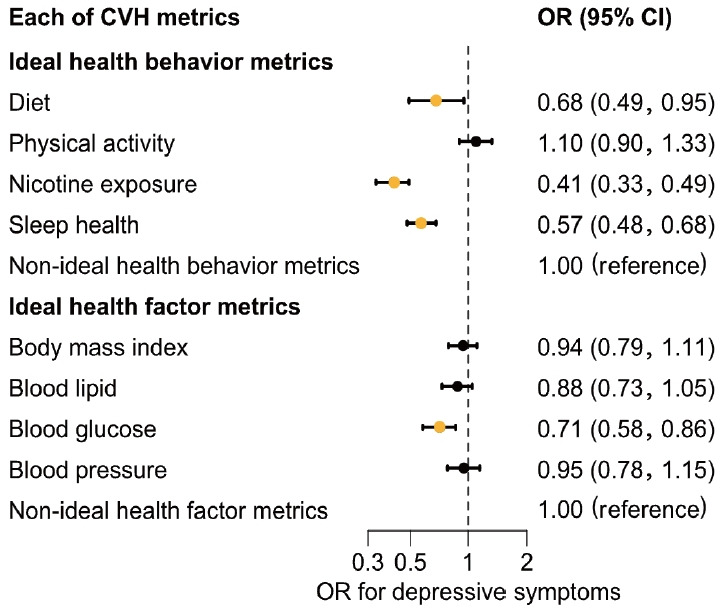
Association between each CVH metric and depressive symptoms. Adjusted for sex, age, income, educational attainment, marital status, and current drinking status. CVH, cardiovascular health; OR, odds ratios; CI, confidence intervals.

Figure 3 shows associations between individual CVH metrics and depressive symptoms. Participants with an ideal diet had lower odds of depressive symptoms compared with those with a non-ideal diet. Similarly, individuals with ideal nicotine exposure had lower odds of depressive symptoms than those with non-ideal nicotine exposure. Participants with ideal sleep health also had lower odds of depressive symptoms compared with those with non-ideal sleep health. Among health factor metrics, individuals with ideal blood glucose had lower odds of depressive symptoms compared with those with non-ideal blood glucose.

**Figure 4.**
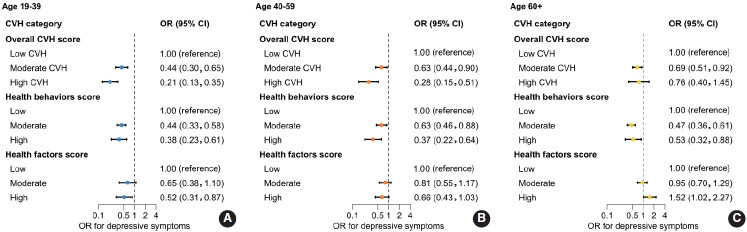
ORs and 95% CIs for the age-stratified (A: 19–39; B: 40–59; and C: ≥60 years) association between CVH scores and depressive symptoms. Overall CVH (by LE8) is divided into two domains: health behaviors (diet, physical activity, nicotine exposure, and sleep health), and health factors (body mass index, blood lipids, blood glucose, and blood pressure). Adjusted for sex, income, educational attainment, marital status, and current drinking status. CVH, cardiovascular health; OR, odds ratios; CI, confidence intervals; LE8, Life’s Essential 8.

Results of sex-stratified and age-stratified analyses are as follows. Compared with participants with a low LE8 score, both male and female with higher LE8 scores had lower odds of depressive symptoms (Supplementary Material 8). Compared with those with non-ideal metrics, female with an ideal diet and male with ideal blood glucose had lower odds of depressive symptoms (Supplementary Material 9). Age-stratified associations between CVH and depressive symptoms are shown in Figure 4. Among participants aged 19–39 years and 40–59 years, those with moderate or high CVH had lower odds of depressive symptoms compared with those with low CVH. Similarly, those with moderate or high health behavior scores had lower odds of depressive symptoms compared with those with low scores. However, among participants aged ≥60 years, high CVH was not significantly associated with depressive symptoms compared with low CVH.

In sensitivity analyses, we evaluated associations between CVH and depressive symptoms by comparing LS7-based and LE8-based models (Supplementary Material 10). We examined the OR for depressive symptoms per 1-point increment in LS7 and per 10-point increment in LE8. The OR for depressive symptoms was 0.84 (95% CI, 0.81 to 0.88) in the LS7 model and 0.72 (95% CI, 0.68 to 0.77) in the LE8 model. The AUC values for LS7 and LE8 were 0.71 (95% CI, 0.69 to 0.73) and 0.72 (95% CI, 0.70 to 0.74), respectively. The AUC for LE8 was slightly higher than for LS7, and the OR for depressive symptoms was lower in the LE8 model.

## DISCUSSION

This study examined the association between CVH measured using LE8 and depressive symptoms. Higher LE8 and health behavior scores were associated with lower odds of depressive symptoms compared with low scores. Ideal diet, non-smoking status, ideal sleep health, and ideal blood glucose were associated with lower odds of depressive symptoms compared with non-ideal metrics. In stratified analyses, higher health behavior scores were associated with lower odds of depressive symptoms across both sexes and all age groups.

A systematic review published in 2022 summarized evidence on associations between LS7-measured CVH and depressive symptoms through May 2021 [7]. Among the 11 studies identified, 5 examined the same exposure and outcome and reported findings consistent with ours [7] (Supplementary Material 11). These studies focused on populations in the United States, China, and Brazil [8,9,32-34]. Across these studies, LS7-measured CVH was inversely associated with depressive symptoms [8,9,32-34]. In 2023, a prospective study involving 6,980 French individuals similarly reported that a higher number of CVH metrics was associated with lower odds of depressive symptoms [35] (Supplementary Material 11).

Our findings are consistent with prior studies that examined associations between LS7 health behavior metrics and depressive symptoms. In a study of United States adults without a mental disorder at baseline, individuals with a greater number of health behavior metrics had lower odds of depressive symptoms than those with the fewest metrics [8]. Therefore, engagement in healthy behaviors was associated with lower odds of depressive symptoms.

Compared with the health behavior score, the health factor score showed ORs in a similar direction for depressive symptoms but did not reach statistical significance. The health factor score—including BMI, blood glucose, blood lipids, and BP—reflects longer-term and cumulative biological status rather than short-term changes, which may provide a more rigorous evaluation for subsequent health outcomes [36]. In contrast, depressive symptoms were assessed over the past 2 weeks, which may create temporal discordance between these measures [29]. In addition, health factors may be influenced by residual confounding, such as medication use and underlying comorbidities, although we adjusted for covariates based on prior studies [7,9]. Future research should consider longitudinal designs that better address temporality and time-varying confounding.

Our findings indicated that ideal diet, non-smoking status, and better sleep health were associated with lower odds of depressive symptoms, consistent with previous studies. A prospective study of 5,110 participants reported that meeting 3–4 diet components was associated with lower odds of depressive symptoms compared with meeting 0–1 components [8]. In addition, intermediate or ideal diet quality was associated with lower odds of depression [33]. Former smokers and never-smokers had 27% and 25% lower odds of depressive symptoms, respectively, compared with current smokers [8]. Smoking may elevate inflammatory responses, increasing levels of C-reactive protein, interleukin-6, and white blood cell counts, which could be associated with depressive symptoms [37].

Sleep health, a newly added LE8 component, was also associated with lower odds of depressive symptoms. Prior prospective studies reported that both short and long sleep durations were associated with an increased risk of depressive symptoms, highlighting sleep duration as an important correlate of mental health outcomes [38]. In a study by Patterson et al. [39], optimal sleep health was associated with a lower prevalence ratio of moderate to severe depression. Persistent sleep restriction may affect neuroendocrine stress pathways, including the autonomic sympathetic- adrenal system and the hypothalamic-pituitary-adrenal (HPA) axis [12]. Alterations such as reduced serotonin receptor activity and dysregulation of the HPA axis have been implicated in depression [12].

Poor blood glucose status measured by LS7 was associated with mild depression compared with ideal blood glucose in a prior study [34]. Similarly, our study found an association between blood glucose and depressive symptoms. Potential mechanisms include the psychological burden of managing diabetes and its complications, lifestyle factors, treatment adherence, diabetes with complications, and HPA axis dysfunction [40-42]. In prior work, diabetes diagnosis showed a direct effect on depression, and frailty mediated the association between diabetes diagnosis and depression [40,42]. Blood glucose and depressive symptoms may exhibit a bidirectional relationship, highlighting the need for longitudinal studies.

Our results showed that participants aged 19–39 years (early adulthood) and 40–59 years (middle age) with moderate or high CVH had lower odds of depressive symptoms compared with those with low CVH. However, among participants aged ≥60 years, high CVH was not statistically significantly associated with lower odds of depressive symptoms. In a prior study, age-stratified analyses showed that intermediate and poor CVH were associated with mild depression across age groups [34]. However, while intermediate and poor CVH were associated with moderate-to-severe depression among individuals aged 20–44 years and 45–64 years, no statistically significant association was observed among those aged ≥65 years [34]. These age-related differences may be related to comorbidity burden and individual and social-environmental factors. Older adults may be more exposed to environments and experiences associated with depressive symptoms than younger adults. With aging, adults experience higher burdens of chronic disease, financial difficulties, loneliness, and spousal loss [43-46]. These cumulative experiences may contribute to differences in depressive symptom reporting and to age-related differences in PHQ-9 sensitivity. Survivor bias may also have influenced our findings if older adults aged >60 years were underrepresented due to mortality, although KNHANES is nationally representative.

This study has several strengths. First, we used nationally representative data, which supports generalizability to the Korean adult population and provides precise estimates under the complex sampling design. Second, our findings add to evidence on associations between CVH and depressive symptoms in Korean and broader Asian populations. Third, we assessed overall CVH using definitions and scoring based on AHA guidelines. Fourth, objective metrics—including BMI, blood lipids, blood glucose, and BP—were measured according to standardized protocols.

This study also has limitations. First, because the design was cross-sectional, causal relationships cannot be inferred. To partially address this limitation, we excluded individuals with physician-diagnosed depression and conducted a sensitivity analysis (Supplementary Material 12); results were consistent with the main analyses. In sensitivity analyses using a lower PHQ-9 cutoff to capture mild depressive symptoms, associations were attenuated (Supplementary Material 13). These findings are consistent with prior prospective studies and support the possibility that better CVH may be associated with fewer depressive symptoms and that bidirectional relationships may exist [7,14,15]. Nevertheless, depressive symptoms may influence health behaviors, and reverse causation cannot be fully excluded. Future longitudinal studies should be designed to address potential bidirectionality and reverse causation. Second, health behavior metrics were self-reported and may be under-reported or over-reported, resulting in misclassification or recall bias. Although health behavior metrics were assessed over short time frames and relied on self-report, prior longitudinal and cohort studies have reported that lower LE8-defined CVH is associated with higher risks of cardiovascular disease [47]. For example, compared with low CVH, intermediate or high CVH was associated with reduced cardiovascular disease-specific mortality among 19,951 United States adults aged 30–79 years [48]. These findings suggest that LE8 may provide a reliable summary of CVH. Nevertheless, longitudinal studies are needed to evaluate associations between LE8-defined CVH and depressive symptoms or depression outcomes. Third, unmeasured confounding (e.g., psychosocial stress, comorbidity, or psychotropic medication use) may remain. Although we adjusted for demographic variables, socioeconomic status, and current drinking status, future studies should consider additional confounders.

In conclusion, higher LE8 and health behavior scores were associated with lower odds of depressive symptoms, whereas no significant association was observed for the health factor score. Among individual components, ideal diet, non-smoking status, ideal sleep health, and ideal blood glucose were associated with lower depressive symptoms. Prospective longitudinal studies are needed to clarify the temporal direction of these associations.

## Figures and Tables

**Figure f5-epih-48-e2026013:**
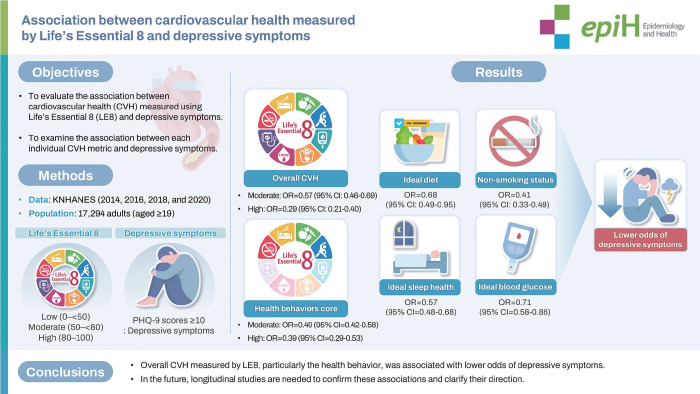


**Table 1.** General characteristics of the study population by depressive symptoms (n=17,294)^1^

Characteristics	No depressive symptoms (PHQ-9<10)	Depressive symptoms (PHQ-9≥10)	p-value
Total	16,356 (95.0)	938 (5.0)	
Sex			<0.001
Male	6,993 (50.8)	257 (34.6)	
Female	9,363 (49.2)	681 (65.4)	
Age (yr)			<0.001
19–39	4,555 (36.1)	293 (43.3)	
40–59	6,185 (41.6)	269 (30.8)	
≥60	5,616 (22.3)	376 (25.9)	
Income (million KRW)			<0.001
<18.4	3,934 (18.6)	443 (39.8)	
18.4-<35.5	4,033 (24.8)	194 (21.5)	
35.5-<58.3	3,933 (26.2)	189 (23.1)	
≥58.3	4,425 (30.5)	111 (15.5)	
Educational attainment (yr)			<0.001
≤6	3,091 (12.6)	303 (21.8)	
7–9	1,667 (8.2)	105 (9.0)	
10–12	5,467 (37.1)	291 (37.1)	
>12	6,125 (42.1)	239 (32.1)	
Marital status			<0.001
Never married	2,673 (23.8)	196 (31.2)	
Married-living together	11,660 (67.0)	484 (48.1)	
Married-separate	97 (0.5)	11 (0.8)	
Divorced, widowed	1,921 (8.7)	246 (19.9)	
Current drinking status			0.007
No	7,676 (41.8)	500 (47.2)	
Yes	8,678 (58.2)	438 (52.8)	
CVH metrics score (0–100)			
Overall score (by LE8)	64.8 (64.4, 65.1)	59.8 (58.7, 61.0)	<0.001
Diet score (by KHEI)	46.6 (45.8, 47.3)	37.0 (34.6, 39.5)	<0.001
Physical activity score	34.2 (33.2, 35.3)	29.9 (26.6, 33.2)	<0.001
Nicotine exposure score	71.8 (71.0, 72.6)	62.7 (59.4, 66.0)	<0.001
Sleep health score	81.5 (81.1, 82.0)	68.4 (65.8, 71.0)	<0.001
Body mass index score	67.8 (67.2, 68.4)	66.6 (63.9, 69.3)	<0.001
Blood lipids score	67.5 (66.9, 68.1)	68.3 (66.1, 70.6)	<0.001
Blood glucose score	78.2 (77.6, 78.7)	74.8 (72.7, 77.0)	<0.001
Blood pressure score	70.4 (69.7, 71.2)	70.9 (68.2, 73.5)	<0.001
CVH category (by LE8 score)			<0.001
Low CVH	2,410 (15.4)	216 (23.4)	
Moderate CVH	11,513 (69.0)	645 (68.2)	
High CVH	2,433 (15.5)	77 (8.4)	

Values are presented as number (weighted %) and mean (95% confidence intervals). PHQ-9, Patient Health Questionnaire-9; KRW, Korean won; CVH, cardiovascular health; KHEI, Korean Healthy Eating Index for Adults; LE8, Life’s Essential 8.

1 The sum of numbers may not apply to the total number in group due to missing values.

## Data Availability

KNHANES data are publicly available at https://knhanes.kdca.go.kr/knhanes/main.do. To obtain the dataset, researchers must complete a straightforward application process through the official website, and data use is permitted only after approval through the site.

## Supplementary materials

Supplementary Material 1. Flow diagram of the study population

Supplementary Material 2. Definition and scoring of Life’s Essential 8 based on the American Heart Association

Supplementary Material 3. Detailed scoring criteria for diet metric using the Korean Healthy Eating Index (KHEI) for adults, developed by the Korea Centers for Disease Control and Prevention

Supplementary Material 4. Sleep health questionnaire used in the Korea National Health and Nutrition Examination Survey

Supplementary Material 5. Detailed measurement of health factors using standardized equipment

Supplementary Material 6. Definition and scoring of Life’s Simple 7 based on the American Heart Association

Supplementary Material 7. Association between cardiovascular health scores and depressive symptoms (multiple imputation data)

Supplementary Material 8. Odds ratios (OR) and 95% Confidence Intervals (CI) for sex-stratified association between cardiovascular health scores and depressive symptoms

Supplementary Material 9. Odds ratios (OR) and 95% Confidence Intervals (CI) for sex-stratified association between each of cardiovascular health metrics and depressive symptoms

Supplementary Material 10. Comparison of ROC curves predicting depressive symptoms using cardiovascular health metrics: Life’s Simple 7 vs. Life’s Essential 8.

Supplementary Material 11. Previous studies on the association between cardiovascular health by Life’s Simple 7 and depressive symptoms.

Supplementary Material 12. Association between cardiovascular health scores and depressive symptoms, excluding participants who had previously been diagnosed with depression (N=16,429)

Supplementary Material 13. Association between cardiovascular health scores and mild depressive symptoms (cut-off≥5)
