# Supplementary material for: Association between cardiovascular health measured by Life’s Essential 8 and depressive symptoms
Source: Epidemiol Health. 2026 Feb 27;48:e2026013. doi: 10.4178/epih.e2026013 (PMC13219981; doi:10.4178/epih.e2026013)
Supplement: Supplementary Material 1. — Flow diagram of the study population [file epih-48-e2026013-Supplementary-1.docx]

**Enrolled Participants**

**(KNHANES 2014, 2016, 2018, and 2020)**

**N=31,051**

**Participants (aged≥19)**

**N=22,680**

**Excluded participants, N=8,371**

1. Aged <19
2. Pregnant women
3. Fasting time <8hours

**Excluded participants, N=5,386**

1. No information on Life's Essential 8
2. No information on PHQ-9

**Final participants**

**N=17,294**

**Supplementary Material 1.** Flow diagram of the study population
