# Supplementary material for: Association between cardiovascular health measured by Life’s Essential 8 and depressive symptoms
Source: Epidemiol Health. 2026 Feb 27;48:e2026013. doi: 10.4178/epih.e2026013 (PMC13219981; doi:10.4178/epih.e2026013)
Supplement: Supplementary Material 2. — Definition and scoring of Life’s Essential 8 based on the American Heart Association [file epih-48-e2026013-Supplementary-2.docx]

**Supplementary Material 2.** Definition and scoring of Life’s Essential 8 based on the American Heart Association

| **Domain** | **CVH metric** | **Definition and details** | **Points** | **Status** |
| --- | --- | --- | --- | --- |
| Health behaviors | 1. Diet | KHEI: 24-hour recall, reference population in KNHANES from 2019 to 2021 | 100 | ≥95th percentile |
|  |  |  | 80 | 75th–<95th percentile |
|  |  |  | 50 | 50th–<75th percentile |
|  |  |  | 25 | 25th–<50th percentile |
|  |  |  | 0 | 1st–<25th percentile |
|  | 2. Physical activity | Moderate or vigorous intensity activity (work and leisure) per week  : one minute of moderate PA per minute  and two minutes of vigorous PA per minute | 100 | ≥150 minutes |
|  |  |  | 90 | 120-<150 minutes |
|  |  |  | 80 | 90-<120 minutes |
|  |  |  | 60 | 60-<90 minutes |
|  |  |  | 40 | 30-<60 minutes |
|  |  |  | 20 | 1-<30 minutes |
|  |  |  | 0 | 0 |
|  | 3. Nicotine exposure | Self-reported use of cigarettes (tobacco), NDS, and secondhand smoke exposure: minus 20 points for living with active indoor smoker in home | 100 | Never smoker |
|  |  |  | 75 | Former smoker, quit ≥5 y |
|  |  |  | 50 | Former smoker, quit 1–<5 y |
|  |  |  | 25 | Former smoker, quit <1 y, or currently using inhaled NDS |
|  |  |  | 0 | Current smoker |
|  | 4. Sleep health | Self-reported average hours of sleep duration | 100 | 7-<9 hours |
|  |  |  | 90 | 9-<10 hours |
|  |  |  | 70 | 6-<7 hours |
|  |  |  | 40 | 5-<6 or ≥10 hours |
|  |  |  | 20 | 4-<5 hours |
|  |  |  | 0 | <4 hours |
| Health factors | 5. Body mass index | Body mass index (kg/m^2^) = body weight (kg) / height squared (m^2^) | 100 | <23 kg/m^2^ |
|  |  |  | 70 | 23.0-<25.0 kg/m^2^ |
|  |  |  | 30 | 25.0-<30.0 kg/m^2^ |
|  |  |  | 15 | 30.0-<35.0 kg/m^2^ |
|  |  |  | 0 | ≥35.0 kg/m^2^ |
|  | 6. Blood lipids | Non-HDL cholesterol (mg/dL)  = TC - HDL-C | 100 | <130 mg/dL |
|  |  |  | 60 | 130-<160 mg/dL |
|  |  |  | 40 | 160-<190 mg/dL |
|  |  |  | 20 | 190-220 mg/dL |
|  |  |  | 0 | ≥220 mg/dL |
|  | 7. Blood glucose | Fasting blood glucose (mg/dL) and HbA1c (%) | 100 | No history of diabetes and FBG <100 (or HbA1c <5.7) |
|  |  |  | 60 | No diabetes and FBG 100–125 (or HbA1c 5.7-<6.5) |
|  |  |  | 40 | Diabetes with HbA1c 6.5-<7.0 |
|  |  |  | 30 | Diabetes with HbA1c 7.0-<8.0 |
|  |  |  | 20 | Diabetes with HbA1c 8.0-<9.0 |
|  |  |  | 10 | Diabetes with HbA1c 9.0-<10.0 |
|  |  |  | 0 | Diabetes with HbA1c ≥10.0 |
|  | 8. Blood pressure | Systolic and diastolic blood pressure (mmHg) | 100 | <120/<80 mmHg |
|  |  |  | 75 | 120-<130/<80 mmHg |
|  |  |  | 50 | 130-<140 or 80-<90 mmHg |
|  |  |  | 25 | 140-<160 or 90-<100 mmHg |
|  |  |  | 0 | ≥160 or ≥100 mmHg |
| Abbreviations: CVH = Cardiovascular health; KHEI = Korean Healthy Eating Index for adults; PA = Physical activity; NDS = Nicotine delivery system; TC = Total cholesterol; HDL = High-density lipoprotein | | | | |
