# Supplementary material for: Association between cardiovascular health measured by Life’s Essential 8 and depressive symptoms
Source: Epidemiol Health. 2026 Feb 27;48:e2026013. doi: 10.4178/epih.e2026013 (PMC13219981; doi:10.4178/epih.e2026013)
Supplement: Supplementary Material 3. — Detailed scoring criteria for diet metric using the Korean Healthy Eating Index (KHEI) for adults, developed by the Korea Centers for Disease Control and Prevention [file epih-48-e2026013-Supplementary-3.docx]

**Supplementary Material 3.** Detailed scoring criteria for diet metric using the Korean Healthy Eating Index (KHEI) for adults, developed by the Korea Centers for Disease Control and Prevention

| **Domain** | **14 components** | **Points** |
| --- | --- | --- |
| **Adequacy (8)** | 1. Breakfast | 10 points |
|  | 2. Mixed grain* | 5 points |
|  | 3. Total fruit* | 5 points |
|  | 4. Fresh fruits* | 5 points |
|  | 5. Total vegetables* | 5 points |
|  | 6. Vegetables (excluding Kimchi and pickled vegetables)* | 5 points |
|  | 7. Meat/fish/eggs and beans | 10 points |
|  | 8. Milk and dairy products | 10 points |
| **Moderation (3)** | 9. Saturated fatty acids | 10 points |
|  | 10. Sodium | 10 points |
|  | 11. Sweets and beverages | 10 points |
| **Energy Balance (3)*** | 12. Carbohydrates | 5 points |
|  | 13. Total fat | 5 points |
|  | 14. Energy | 5 points |
| **Total** |  | 100 points |

*5 points based on the 2015 Korean Dietary Reference Intake guidelines

The reference population for quantiles of KHEI used KNHANES data from 2016 to 2018.

Abbreviations: KHEI = Korean Healthy Eating Index for adults; KNHANES = Korea National Health and Nutrition Examination Survey
