# Supplementary material for: Association between cardiovascular health measured by Life’s Essential 8 and depressive symptoms
Source: Epidemiol Health. 2026 Feb 27;48:e2026013. doi: 10.4178/epih.e2026013 (PMC13219981; doi:10.4178/epih.e2026013)
Supplement: Supplementary Material 4. — Sleep health questionnaire used in the Korea National Health and Nutrition Examination Survey [file epih-48-e2026013-Supplementary-4.docx]

**Supplementary Material 4.** Sleep health questionnaire used in the Korea National Health and Nutrition Examination Survey

| **Health Behavior Metric** | **Year** | **Questionnaire** |
| --- | --- | --- |
| Sleep health | 2014, 2020 | “How many hours do you usually sleep per day?” |
|  | 2016, 2018* | What time do you usually go to bed and wake up on weekdays? |
|  |  | What time do you usually go to bed and wake up on weekends? |
| *Average daily sleep duration using the sleep hours on weekdays and weekends. | | |
