# Supplementary material for: Association between cardiovascular health measured by Life’s Essential 8 and depressive symptoms
Source: Epidemiol Health. 2026 Feb 27;48:e2026013. doi: 10.4178/epih.e2026013 (PMC13219981; doi:10.4178/epih.e2026013)
Supplement: Supplementary Material 5. — Detailed measurement of health factors using standardized equipment [file epih-48-e2026013-Supplementary-5.docx]

Supplementary Material 5. Detailed measurement of health factors using standardized equipment

| **Measurement** | **Equipment (Manufacturer/Country)** | **Year** |
| --- | --- | --- |
| Total cholesterol, HDL-C | Hitachi Automatic Analyzer 7600 (Hitachi, Japan) | 2014, 2016, 2018 |
| Total cholesterol, HDL-C | Labospect 008AS (Hitachi, Japan) | 2020 |
| Fasting blood glucose | Hitachi Automatic Analyzer 7600 (Hitachi, Japan) | 2014, 2016, 2018, 2020 |
| HbA1c | Tosoh G8 (Tosoh, Japan) | 2014, 2016, 2018, 2020 |
| Blood pressure | Mercury sphygmomanometer (Baumanometer, Baum, USA) | 2014, 2016, 2018 |
| Blood pressure | Mercury-free sphygmomanometer (Greenlight 300) | 2020 |

Abbreviations: HDL = High-density lipoprotein;
