# Supplementary material for: Association between cardiovascular health measured by Life’s Essential 8 and depressive symptoms
Source: Epidemiol Health. 2026 Feb 27;48:e2026013. doi: 10.4178/epih.e2026013 (PMC13219981; doi:10.4178/epih.e2026013)
Supplement: Supplementary Material 6. — Definition and scoring of Life’s Simple 7 based on the American Heart Association [file epih-48-e2026013-Supplementary-6.docx]

**Supplementary Material 6.** Definition and scoring of Life’s Simple 7 based on the American Heart Association

| **Domain** | **Metrics** | **Definition and details** | **Points** |
| --- | --- | --- | --- |
| Health behavior | 1. Diet | 0-<50 | 0 (Poor) |
|  |  | 50-<80 | 1 (Intermediate) |
|  |  | ≥80 | 2 (Ideal) |
|  | 2. Physical activity | No physical activity | 0 (Poor) |
|  |  | Moderate intensity (1-<150 min/wk) or vigorous intensity (1-<75 min/wk) or moderate to vigorous (1-<150 min/wk) | 1 (Intermediate) |
|  |  | Moderate intensity (≥150 min/wk) or vigorous intensity (≥75min/wk) or moderate to vigorous (≥150 min/wk) | 2 (Ideal) |
|  | 3. Smoking | Current smoker | 0 (Poor) |
|  |  | Former smoker, quit≤12 month | 1 (Intermediate) |
|  |  | Never smoker or quit>12 month | 2 (Ideal) |
|  | 4. Body mass index | ≥30 kg/m2 | 0 (Poor) |
|  |  | 25-<30kg/m2 | 1 (Intermediate) |
|  |  | <25 kg/m2 | 2 (Ideal) |
| Health factor | 5. Total cholesterol | ≥240 mg/dL | 0 (Poor) |
|  |  | 200-<240 mg/dL or treated to goal | 1 (Intermediate) |
|  |  | <200 mg/dL | 2 (Ideal) |
|  | 6. Fasting plasma glucose | ≥ 126 mg/dL | 0 (Poor) |
|  |  | 100-<126 mg/dL or treated to goal | 1 (Intermediate) |
|  |  | <100 mg/dL | 2 (Ideal) |
|  | 7. Blood pressure | SBP≥140 or DBP≥90 mmHg | 0 (Poor) |
|  |  | SBP 120-<140 or DBP 80-<90 mmHg or treated to goal | 1 (Intermediate) |
|  |  | <120 / <80 mmHg | 2 (Ideal) |
| Diet was assessed using the Korean Healthy Eating Index for adults (KHEI). | | | |
| Life's Simple 7 score is the sum of the two domains of health behaviors (diet, physical activity, smoking, and body mass index) and health factors (total cholesterol, fasting plasma glucose, and blood pressure) | | | |
| Abbreviations: LS7 = Life's Simple 7; SBP = Systolic Blood pressure; DBP = Diastolic Blood Pressure | | | |
