# Supplementary material for: Association between cardiovascular health measured by Life’s Essential 8 and depressive symptoms
Source: Epidemiol Health. 2026 Feb 27;48:e2026013. doi: 10.4178/epih.e2026013 (PMC13219981; doi:10.4178/epih.e2026013)
Supplement: Supplementary Material 7. — Association between cardiovascular health scores and depressive symptoms (multiple imputation data) [file epih-48-e2026013-Supplementary-7.docx]

**Supplementary Material 7.** Association between cardiovascular health scores and depressive symptoms (multiple imputation data)

|  | | **Depressive symptoms** | |
| --- | --- | --- | --- |
|  |  | **OR** | **(95% CI)** |
| **Categorical CVH status (by LE8 score)** | | |  |
| Low CVH | | Reference | |
| Moderate CVH | | 0.56 | (0.46–0.69) |
| High CVH | | 0.29 | (0.21–0.40) |
|  | **Health behaviors score** | |  |
|  | Low | Reference | |
|  | Moderate | 0.49 | (0.42–0.58) |
|  | High | 0.40 | (0.29–0.53) |
|  | **Health factors score** | |  |
|  | Low | Reference | |
|  | Moderate | 0.80 | (0.64–1.01) |
|  | High | 0.74 | (0.57–0.96) |

Adjusted for age, sex income, educational attainment, marital status, and current drinking status.

Overall CVH score, health behaviors score, and health factors score are categorized as low (0-<50), moderate (50-<80), and high (80-<100).

Overall CVH (by LE8) is divided into 2 domains: health behaviors (diet, physical activity, nicotine exposure, and sleep health), and health factors (body mass index, blood lipids, blood glucose, and blood pressure)

Abbreviations: Cardiovascular health = CVH; Life's essential 8 = LE8
