# Supplementary material for: Association between cardiovascular health measured by Life’s Essential 8 and depressive symptoms
Source: Epidemiol Health. 2026 Feb 27;48:e2026013. doi: 10.4178/epih.e2026013 (PMC13219981; doi:10.4178/epih.e2026013)
Supplement: Supplementary Material 8. — Odds ratios (OR) and 95% Confidence Intervals (CI) for sex-stratified association between cardiovascular health scores and depressive symptoms [file epih-48-e2026013-Supplementary-8.docx]

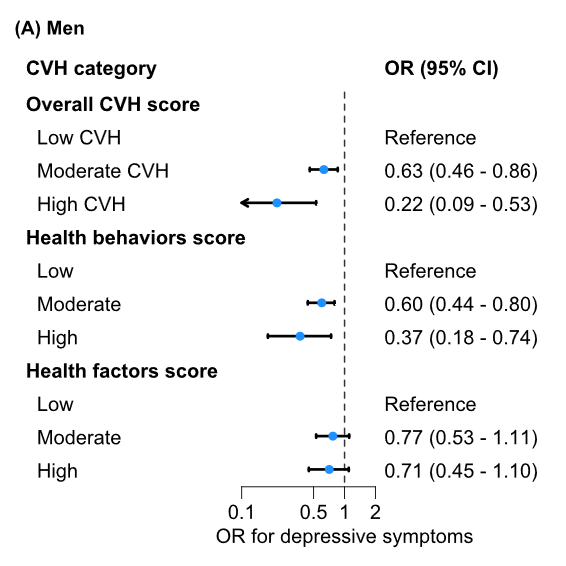

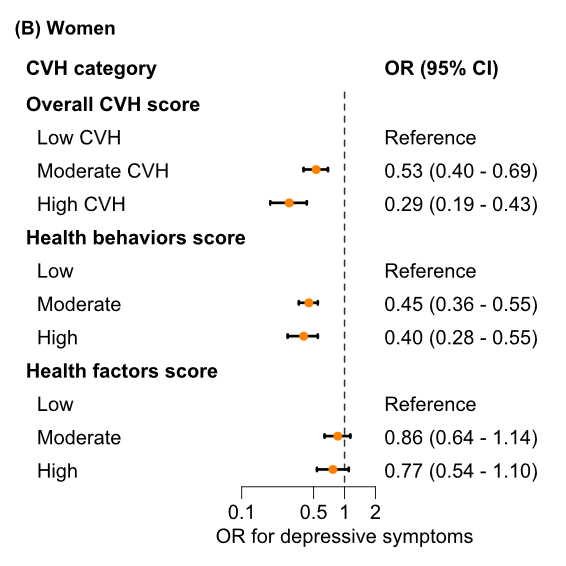


**Supplementary Material 8.** Odds ratios (OR) and 95% Confidence Intervals (CI) for sex-stratified association between cardiovascular health scores and depressive symptoms

Overall CVH (by LE8) is divided into two domains: health behaviors (diet, physical activity, nicotine exposure, and sleep health), and health factors (body mass index, blood lipids, blood glucose, and blood pressure)

Adjusted for age, income, educational attainment, marital status, and current drinking status

Abbreviations: Cardiovascular health = CVH;
