# Supplementary material for: Association between cardiovascular health measured by Life’s Essential 8 and depressive symptoms
Source: Epidemiol Health. 2026 Feb 27;48:e2026013. doi: 10.4178/epih.e2026013 (PMC13219981; doi:10.4178/epih.e2026013)
Supplement: Supplementary Material 9. — Odds ratios (OR) and 95% Confidence Intervals (CI) for sex-stratified association between each of cardiovascular health metrics and depressive symptoms [file epih-48-e2026013-Supplementary-9.docx]

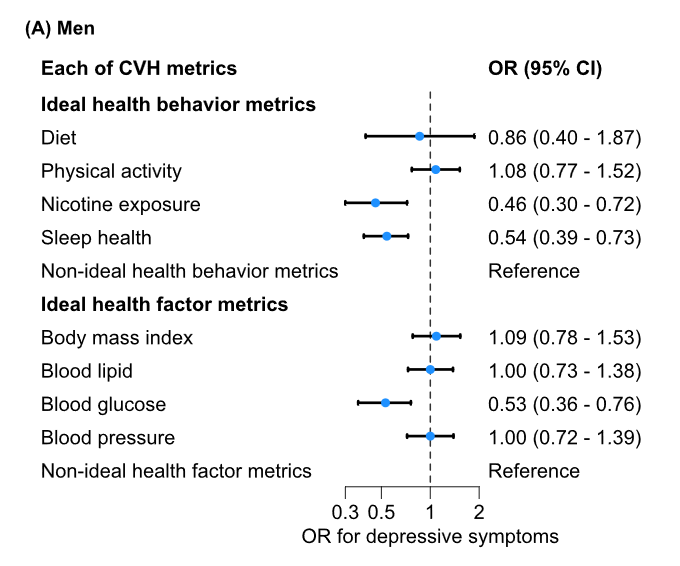

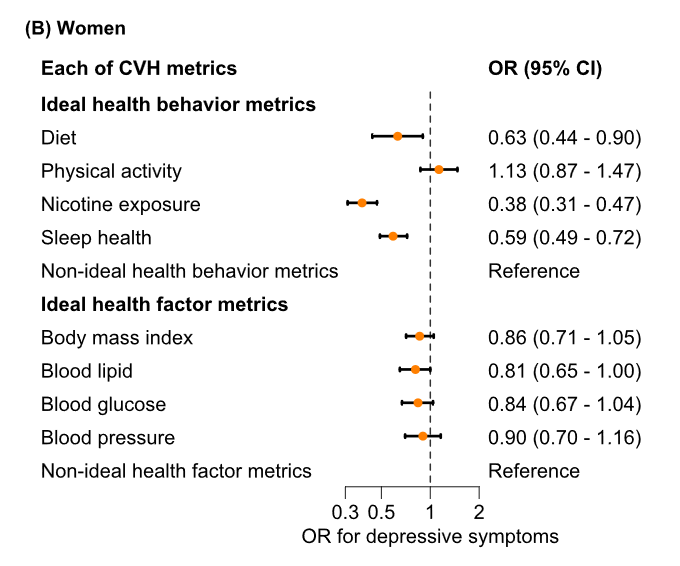


Supplementary Material 9. Odds ratios (OR) and 95% Confidence Intervals (CI) for sex-stratified association between each of cardiovascular health metrics and depressive symptoms

Adjusted for age, income, educational attainment, marital status, and current drinking status

Abbreviations: Cardiovascular health=CVH;
