# Supplementary material for: Association between cardiovascular health measured by Life’s Essential 8 and depressive symptoms
Source: Epidemiol Health. 2026 Feb 27;48:e2026013. doi: 10.4178/epih.e2026013 (PMC13219981; doi:10.4178/epih.e2026013)
Supplement: Supplementary Material 10. — Comparison of ROC curves predicting depressive symptoms using cardiovascular health metrics: Life’s Simple 7 vs. Life’s Essential 8. [file epih-48-e2026013-Supplementary-10.docx]

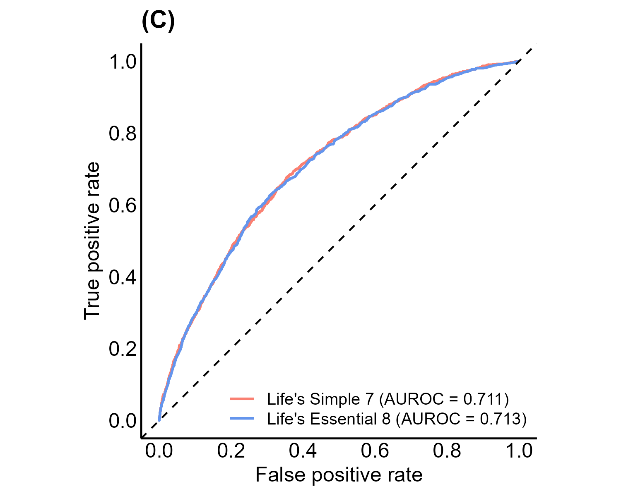

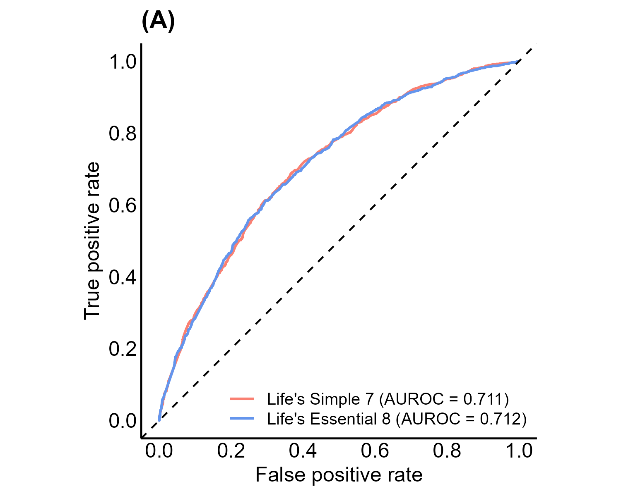

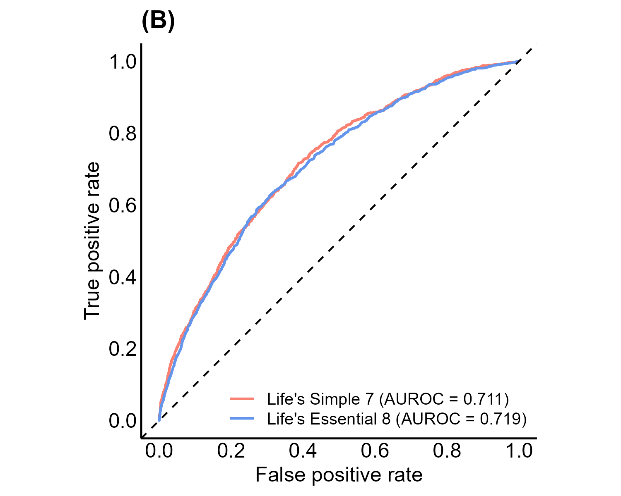


**Supplementary Material 10.** Comparison of ROC curves predicting depressive symptoms using cardiovascular health metrics: Life’s Simple 7 vs. Life’s Essential 8.

(A) Categorical cardiovascular health measured by Life’s Simple 7 (AUROC=0.711, 95% CI=0.695–0.728) and Life’s Essential 8 (AUROC=0.712, 95% CI=0.695–0.728).

(B) Continuous cardiovascular health measured by Life’s Simple 7 (AUROC=, 0.711, 95% CI=0.694–0.727) and Life’s Essential 8 (AUROC=0.719, 95% CI=0.703–0.735)

(C) Continuous cardiovascular health excluding sleep, measured by Life’s Simple 7 (AUROC=0.711, 95% CI=0.694–0.727) and Life’s Essential 8 (AUROC=0.713, 95% CI=0.696–0.729).

Abbreviations: AUROC=the area under the receiver operating characteristic curves; ROC=Receiver operating characteristic; CI=Confidence interval;

*Continuous overall cardiovascular health measured by Life’s Essential 8: per 10 increases
