# Supplementary material for: Association between cardiovascular health measured by Life’s Essential 8 and depressive symptoms
Source: Epidemiol Health. 2026 Feb 27;48:e2026013. doi: 10.4178/epih.e2026013 (PMC13219981; doi:10.4178/epih.e2026013)
Supplement: Supplementary Material 11. — Previous studies on the association between cardiovascular health by Life’s Simple 7 and depressive symptoms. [file epih-48-e2026013-Supplementary-11.docx]

**Supplementary Material 11.** Previous studies on the association between cardiovascular health by Life’s Simple 7 and depressive symptoms.

| **No.** | **Author** | **Year** | **Study design** | **Population** | **N** | **Exposure** | **Outcome** |
| --- | --- | --- | --- | --- | --- | --- | --- |
| **The systematic review of the association between CVH measured by LS7 and depressive symptoms** | | | | | | | |
| 1 | España-Romero et al. | 2013 | Prospective study | United States | 5,110 | No. of CVH metrics | Depression (CES-D) |
| 2 | Zeng et al | 2013 | Cross-sectional study | China | 9,962 | No. of CVH metrics | Depression (DASS-21) |
| 3 | Li et al | 2015 | Cross-sectional study | China | 6,851 | CVH score | Depression (CES-D) |
| 4 | Brunoni et al | 2019 | Prospective study | Brazil | 9,214 | CVH score | Depression (CIS-R) |
| 5 | Zhang et al | 2019 | Cross-sectional study | United States | 14,561 | CVH score | Depression (PHQ-9) |
| **A recent study of the association between CVH measured by LS7 and depressive symptoms** | | | | | | | |
| 6 | van Sloten et al | 2023 | Prospective study | France | 6,980 | No. of CVH metrics | Depressive symptoms (CES-D) |

Abbreviations: CVH = Cardiovascular health; LS7 = Life’s Simple 7; CESD = Center for Epidemiologic Studies Depression; DASS-21 = Depression Anxiety Stress Scales-21; CIS-R = Clinical interview schedule-revised; PHQ-9 = Patient health questionnaire-9;
