# Supplementary material for: Association between cardiovascular health measured by Life’s Essential 8 and depressive symptoms
Source: Epidemiol Health. 2026 Feb 27;48:e2026013. doi: 10.4178/epih.e2026013 (PMC13219981; doi:10.4178/epih.e2026013)
Supplement: Supplementary Material 12. — Association between cardiovascular health scores and depressive symptoms, excluding participants who had previously been diagnosed with depression (N=16,429) [file epih-48-e2026013-Supplementary-12.docx]

**Supplementary Material 12.** Association between cardiovascular health scores and depressive symptoms, excluding participants who had previously been diagnosed with depression (N=16,429)

| ` | | **N** |  | **Case (%)** | **Model 1** | **Model 2** | **Model 3** | **Model 4** |
| --- | --- | --- | --- | --- | --- | --- | --- | --- |
|  |  |  |  |  | **OR (95% CI)** | **OR (95% CI)** | **OR (95% CI)** | **OR (95% CI)** |
| **Categorical CVH status (by LE8 score)** | | | | | | | | |
| Low CVH | | 2,481 |  | 154 (6.2) | Reference | Reference | Reference | Reference |
| Moderate CVH | | 11,526 |  | 495 (4.3) | 0.63 (0.51–0.79) | 0.49 (0.39–0.61) | 0.55 (0.44–0.69) | 0.55 (0.44–0.70) |
| High CVH | | 2,422 |  | 63 (2.6) | 0.39 (0.28–0.55) | 0.25 (0.18–0.36) | 0.31 (0.22–0.45) | 0.31 (0.22–0.45) |
|  | **Health behaviors score** | | | | | | | |
|  | Low | 4,617 |  | 309 (6.7) | Reference | Reference | Reference | Reference |
|  | Moderate | 9,386 |  | 338 (3.6) | 0.54 (0.45–0.65) | 0.45 (0.37–0.55) | 0.50 (0.41–0.60) | 0.50 (0.41–0.60) |
|  | High | 2,426 |  | 65 (2.7) | 0.39 (0.28–0.53) | 0.33 (0.24–0.45) | 0.41 (0.30–0.57) | 0.41 (0.30–0.57) |
|  | **Health factors score** | | | | | | | |
|  | Low | 2,700 |  | 123 (4.6) | Reference | Reference | Reference | Reference |
|  | Moderate | 7,868 |  | 335 (4.3) | 1.01 (0.71–1.42) | 0.98 (0.69–1.38) | 1.07 (0.75–1.51) | 1.06 (0.75–1.50) |
|  | High | 5,861 |  | 254 (4.3) | 1.02 (0.73–1.42) | 0.84 (0.59–1.20) | 0.98 (0.68–1.42) | 0.98 (0.68–1.41) |
| Model 1: Unadjusted model | | | | | | | | |
| Model 2: Adjusted for age and sex | | | | | | | | |
| Model 3: Model 2 + Adjusted for income, educational attainment and marital status. | | | | | | | | |
| Model 4: Model 3 + Adjusted for current drinking status | | | | | | | | |
| Overall CVH score, health behaviors score, and health factors score are categorized as low (0-<50), moderate (50-<80), and high (80-<100). | | | | | | | | |
| Overall CVH (by LE8) is divided into 2 domains: health behaviors (diet, physical activity, nicotine exposure, and sleep health), and health factors (body mass index, blood lipids, blood glucose, and blood pressure) | | | | | | | | |
| Abbreviations: Cardiovascular health = CVH; Life's essential 8 = LE8 | | | | | | | | |
