# Supplementary material for: Association between cardiovascular health measured by Life’s Essential 8 and depressive symptoms
Source: Epidemiol Health. 2026 Feb 27;48:e2026013. doi: 10.4178/epih.e2026013 (PMC13219981; doi:10.4178/epih.e2026013)
Supplement: Supplementary Material 13. — Association between cardiovascular health scores and mild depressive symptoms (cut-off≥5) [file epih-48-e2026013-Supplementary-13.docx]

**Supplementary Material 13.** Association between cardiovascular health scores and mild depressive symptoms (cut-off≥5)

|  | | **N** |  | **Case (%)** | **Mild depressive symptoms** |
| --- | --- | --- | --- | --- | --- |
|  |  |  |  |  | **OR (95% CI)** |
| **Categorical CVH status (by LE8 score)** | | | | | |
| Low CVH | | 2,626 |  | 610 (2.3) | Reference |
| Moderate CVH | | 12,158 |  | 2,306 (19.0) | 0.71 (0.63–0.81) |
| High CVH | | 2,510 |  | 422 (16.8) | 0.55 (0.46–0.66) |
|  | **Health behaviors score** | | | | |
|  | Low | 4,895 |  | 1,210 (24.2) | Reference |
|  | Moderate | 9,873 |  | 1,776 (18.0) | 0.63 (0.57–0.70) |
|  | High | 2,526 |  | 352 (13.9) | 0.51 (0.44–0.60) |
|  | **Health factors score** | | | | |
|  | Low | 2,848 |  | 533 (18.7) | Reference |
|  | Moderate | 8,313 |  | 1,537 (18.5) | 0.92 (0.80–1.06) |
|  | High | 6,133 |  | 1,268 (20.7) | 0.91 (0.79–1.05) |

Adjusted for sex, age, income, educational attainment, marital status, and current drinking status

Overall CVH score, health behaviors score, and health factors score are categorized as low (0-<50), moderate (50-<80), and high (80-<100).

Overall CVH (by LE8) is divided into 2 domains: health behaviors (diet, physical activity, nicotine exposure, and sleep health), and health factors (body mass index, blood lipids, blood glucose, and blood pressure)

Abbreviations: Cardiovascular health, CVH; Life's essential 8, LE8; Odds ratios, OR; Confidence intervals, CI;
